# An Improved Method for the Synthesis of Butein Using SOCl_2_/EtOH as Catalyst and Deciphering Its Inhibition Mechanism on Xanthine Oxidase

**DOI:** 10.3390/molecules24101948

**Published:** 2019-05-21

**Authors:** Yu-Xue Hou, Shi-Wei Sun, Yang Liu, Yan Li, Xiao-Hong Liu, Wei Wang, Shuang Zhang, Wei Wang

**Affiliations:** Department of Natural Medicine and Pharmacognosy, School of Pharmacy, Qingdao University, Qingdao 260021, China; hyx19931008@163.com (Y.-X.H.); sunsw@qdu.edu.cn (S.-W.S.); buckuper@163.com (Y.L.); liyanyaohua@126.com (Y.L.); liuxiaohong1043@163.com (X.-H.L.); justwangwade@126.com (W.W.); qdeduzhangshuang@163.com (S.Z.)

**Keywords:** butein, SOCl_2_/EtOH, xanthine oxidase, inhibitory mechanism, kinetic analysis, florescence spectroscopy, molecular docking

## Abstract

Butein (3,4,2′,4′-tetrahydroxychalcone) belongs to the chalcone family of flavonoids and possesses various biological activities. In this study, butein was synthesized through aldol condensation catalyzed by thionyl chloride (SOCl_2_)/ethyl alcohol (EtOH) for the first time. The optimal reaction conditions including the molar ratio of reactants, the dosage of catalyst, and the reaction time on the yield of product were investigated, and the straightforward strategy assembles the yield of butein up to 88%. Butein has been found to inhibit xanthine oxidase (XO) activity. Herein, the inhibitory mechanism of butein against XO was discussed in aspects of inhibition kinetic, fluorescence titration, synchronous fluorescence spectroscopy, and molecular docking. The inhibition kinetic analysis showed that butein possessed a stronger inhibition on XO in an irreversible competitive manner with IC_50_ value of 2.93 × 10^−6^ mol L^−1^. The results of fluorescence titrations and synchronous fluorescence spectroscopy indicated that butein was able to interact with XO at one binding site, and the fluorophores of XO were placed in a more hydrophobic environment with the addition of butein. Subsequently, the result of molecular docking between butein and XO protein revealed that butein formed hydrogen bonding with the amino acid residues located in the hydrophobic cavity of XO. All the results suggested that the inhibitory mechanism of butein on XO may be the insertion of butein into the active site occupying the catalytic center of XO to avoid the entrance of xanthine and inducing conformational changes in XO.

## 1. Introduction

Hyperuricemia characterized by an elevated level of uric acid in human blood is a critical cause of gout [1,2,3]. It can result in the deposition of monosodium urate crystals in joint and soft tissue, with accompanying inflammation and degenerative consequences [4,5]. Currently, the increasing clinical reports have demonstrated that hyperuricemia is associated with some other diseases such as cardiovascular disorders, renal dysfunction, obesity, diabetes, hyperlipidemia, cancer, hypertension, and metabolic syndrome [6]. Thus, maintaining uric acid at a normal level becomes an important therapy for prevention of gout and related disorders. Xanthine oxidase (XO), with a function of catalyzing hypoxanthine and xanthine to uric acid, is a pivotal enzyme in purine metabolism. Structurally, XO is a homodimer with each monomer consisted of the molybdopterin (Mo-pt), the flavin adenine dinucleotide (FAD) and two distinct [2Fe–2S] centers [7]. Although a series of promising alternative treatments of hyperuricemia including the utilization of interleukin-1 inhibitors, recombinant uricase therapy, or the targeting of renal urate transporters have been respectively investigated [8,9,10], XO inhibitors remain a primary therapy owing to the fundamental inhibition of uric acid production. For decades, allopurinol, a purine analogue, has been widely applied to manage hyperuricemia as a potent XO inhibitor. While clearly effective, only about 40% of patients are able to meet treatment goals via allopurinol, and it occasionally causes Stevens Johnson syndrome, which may be fatal [11]. Febuxostat, a non-purine XO inhibitor, is efficacious as a second-line therapy in lowering serum uric acid levels in patients with gout [12]. Unfortunately, mild adverse reactions of febuxostat including musculoskeletal symptoms, abnormal liver-function values, diarrhea, and headache are exhibited, causing the Food and Drug Administration to require a cautionary statement on the drug insert [13,14]. Accordingly, the search for XO inhibitors with fewer side effects than allopurinol and febuxostat is highly warranted.

Early studies have reported the XO inhibitory ability of butein (3,4,2′,4′-tetrahydroxychalcone) belonging to the chalcone family of flavonoids, however, the inhibitory mechanism of butein against XO was unclear until now [15,16]. Therefore, clarifying the inhibitory mechanism of butein on XO activity may provide new insights into the application of butein as a XO inhibitor. Although butein has been isolated from several plants, such as *Butea monosperma*, *Dalbergia odorifera*, *Semecarpus anacardium*, and *Toxicodendron vernicifluum* [17], the preparation of butein from natural plants suffers from drawbacks such as tedious extraction and time-consuming isolation procedures, consumption of large amounts of organic solvents, and poor yield. The main method for the synthesis of butein involves Claisen-Schmidt condensation between 2,4-dihydroxyacetophenone and 3,4-dihydroxybenzaldehyde in the present of basic catalyst [18]. Nonetheless, the issues of complex process and long reaction time are exposed due to the protection and deprotection of hydroxyl groups [16]. Comparing with the basic catalyst, the problem of low productivity was revealed via the adoption of the typical acidic catalyst [19]. Fortunately, thionyl chloride (SOCl_2_)/ethyl alcohol (EtOH) as a novel acidic catalyst for the synthesis of butein was first developed to compensate the shortcoming in this paper, and the optimized reaction condition was established. Subsequently, the inhibitory mechanism of butein against XO was further explored using inhibition kinetic, fluorescence titration, synchronous fluorescence spectroscopy, and molecular docking.

## 2. Results and Discussion

### 2.1. Synthesis of Butein

**Scheme 1:**
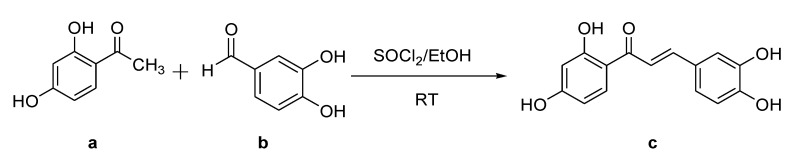
Synthesis of butein.

As outlined in Scheme 1, butein (**c**) was prepared via aldol condensation catalyzed by SOCl_2_ in ethyl alcohol between 2,4-dihydroxyacetophenone (**a**) and 3,4-dihydroxybenzaldehyde (**b**). Although the SOCl_2_/EtOH employed to synthesis chalcones has already been described [20,21], butein was synthesized in this study using SOCl_2_/EtOH as a catalyst in one step for the first time and the protection of the hydroxyl groups were avoided. The reaction mixture was extracted by ethyl acetate to obtain the crude product. Pure butein was purified by reversed-phase preparative HPLC and structural elucidation was identified by MS and NMR spectra. In order to further improve the synthetic reaction efficiency of butein, we studied the influence of various parameters of the reaction such as the molar ratio of reactants, the dosage of catalyst, and reaction time. The overall yield of butein was then measured by HPLC. Firstly, the concentration of SOCl_2_ in ethyl alcohol was critical in aldol condensation. The reactants **a** and **b** (1:1, molar ratio) with different concentrations of SOCl_2_ (0.25, 0.5, 1, 1.5, 2.0 mol L^−1^) were evaluated for the synthetic reaction capacity. As depicted in Table 1, the yields increased quickly among the concentrations of SOCl_2_ from 0.25 mol L^−1^ to 1 mol L^−1^ (entry 1–3), and declined suddenly when the concentrations of SOCl_2_ exceeded 1 mol L^−1^ (entry 4–5). The yields of high catalyst concentration (2.0 mol L^−1^) and low catalyst concentration (0.25 mol L^−1^) were only 9% and 2%, respectively. The highest yield of butein (85%) was afforded when the concentration of catalyst was 1 mol L^−1^, and thus was chosen for further study. Secondly, the molar ratio of two reactants was also investigated. As can be seen in Table 1, the molar ratio of **a** and **b** increasing from 1:0.6 to 1:1 would lead to the increased of yields (entry 6–7). However, the yields decreased with the molar ratio increasing from 1:1 to 1:1.4 (entry 8–9). Therefore, the molar ratio of two reactants of 1:1 (entry 3) was recommended in our case. Besides, the yield of butein was not significantly increased at the longer reaction time. To our delight, the yields could reach 88% within 1 h (entry 10–12), which extremely saved the preparation time. On the basis of above results, a sets of parameters to synthesize butein was proposed: Reaction was carried out at SOCl_2_ 1 mol L^−1^, under room temperature for 1 h, and the molar ratio of **a** and **b** is 1:1 (entry 11).

### 2.2. Inhibition of Butein on XO Activity

**Figure 1:**
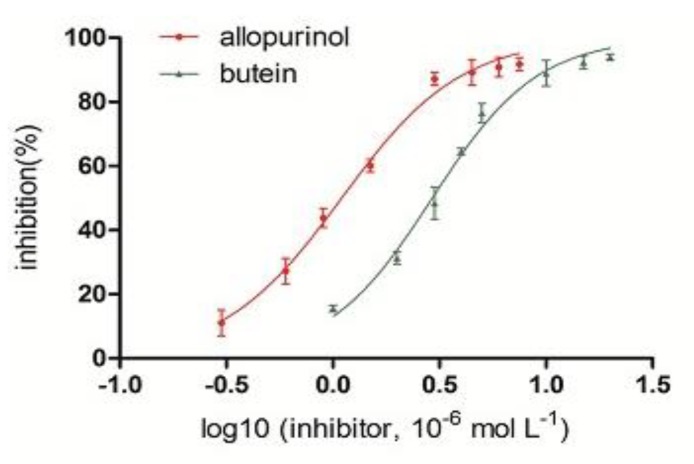
Inhibitory effect of butein on xanthine oxidase (XO) activity (pH = 7.5, T = 298 K). The final concentrations of xanthine and XO were 300 × 10^−6^ mol L^−1^ and 0.024 U mL^−1^, respectively. Each value represents means (±) standard deviation (*n* = 3).

The inhibitory effect of butein on XO activity was evaluated by the formation of uric acid using xanthine as the substrate. As shown in Figure 1, the activity of XO was effectively inhibited by butein; the inhibition rate increased significantly with increasing concentration of the inhibitor. The IC_50_ value of butein was determined to be 2.93 × 10^−6^ mol L^−1^, which was slightly higher than that of allopurinol (1.10 × 10^−6^ mol L^−1^). This IC_50_ value of butein was in keeping with the result of previous report on XO inhibitory activity [16]. The results indicated that butein was an effective XO inhibitor compared to positive control allopurinol, as a result of the presence of carbonyl group and four hydroxyl groups on two phenyl rings [22]. In the previous research, chalcones 3,4,2′,5′-tetrahydroxychalcone and 3,4,2′,6′-tetrahydroxychalcone with a similar structure as butein have shown weaker inhibition on XO with IC_50_ values of (17 ± 8) × 10^−6^ mol L^−1^ and (35 ± 10) × 10^−6^ mol L^−1^, respectively [16]. The presence of hydroxyl groups at C (2′) and C (4′) on ring B showed stronger activity than those with the equivalent substitutes but located at *para-* position on ring B at C (2′) and C (5′) or *meta-* position at C (2′) and C (6′) may be contributes to an increment in the stabilization of the aromatic ring due to induce effect [22,23]. Similarly, compound 2,4,2′,5′-tetrahydroxychalcone with two hydroxyl groups located at the *meta-* position on ring A at C (2) and C (4) showed weaker inhibition activity (16.3 × 10^−6^ mol L^−1^) than butein [22]. That may be because the higher polarizability enhances the attractive dispersion interactions with an aromatic residue of enzyme binding site through π-π stacking interactions [24,25]. Thus, the potential inhibitory mechanism of butein on XO is worthy of further investigation.

### 2.3. Irreversibility 

**Figure 2:**
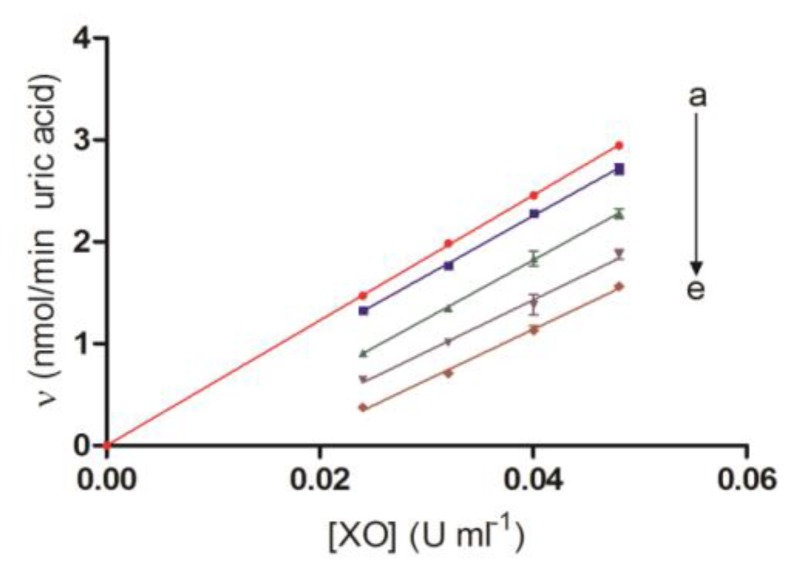
Plots of *ν* versus [XO], c (xanthine) = 300 × 10^−6^ mol L^−1^, c(butein) = 0, 1, 2, 3 and 4 × 10^−6^ mol L^−1^ for curves a → e, respectively. Each value represents means (±) standard deviation (*n* = 3).

The inhibition of XO by diverse concentrations of butein was illustrated in Figure 2. The plots of the velocity versus at various concentrations of XO were constructed to confirm the irreversibility of butein mediated inhibition. The plots offered a series of parallel straight lines and the slopes of the lines were unchanged with the increasing concentrations of butein, which indicated that butein was an irreversible inhibitor on XO. To the best of our knowledge, most of flavonoids were reported as a reversible inhibitor on XO [26,27,28]. The different inhibition type of butein on XO prompts us to further understand the potential mechanism between butein and XO. 

### 2.4. Inhibition Kinetic Analysis 

**Figure 3:**
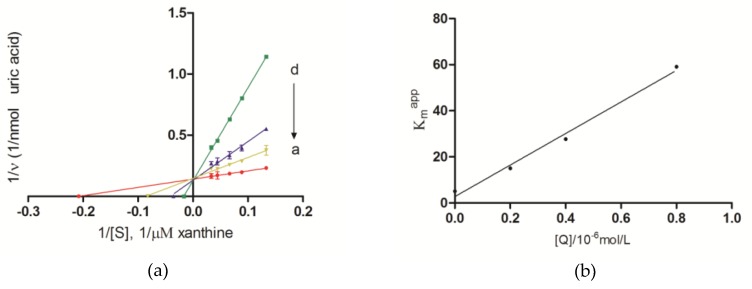
(**a**) Lineweaver–Burk plots. c(XO)=0.08 U mL^−1^, c(butein) = 0.8, 0.4, 0.2 × 10^−6^ mol L^−1^ and 0 for curves d → a, respectively; (**b**) the *K*_m_^app^ versus [butein]. Each value represents means (±) standard deviation (*n* = 3).

Enzyme inhibition kinetic experiments were carried out to further characterize the binding type of butein against XO by analyzing the data from Lineweaver–Burk plots. As shown in Figure 3a, the vertical axis intercept (1/*V*_max_) of the double-reciprocal Lineweaver–Burk plots remained the same and horizontal axis intercept (−1/*K*_m_) increased with accumulating concentrations of butein, suggesting that butein is a competitive inhibitor. Figure 3b exhibits good linearity between *K*_m_^app^ and the relative concentration of butein, signifying that butein bound in a single class of inhibition sites on XO. The inhibition kinetic analysis can be inferred that butein bound to the same active site of XO as xanthine and competed with xanthine during catalysis, just as the allopurinol under the uniform experimental conditions [29]. The value of *K*_i_ (inhibition constant) was determined to be 0.46 × 10^−7^ mol L^−1^.

### 2.5. Fluorescence Quenching of XO upon Butein

**Figure 4:**
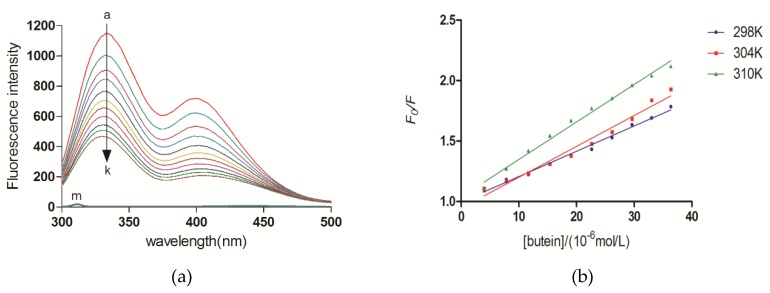
(**a**) c(XO) = 0.1 U mL^−1^ and c(butein) = 0, 3.96, 7.84, 11.65, 15.39, 19.05, 22.64, 26.17, 29.63, 33.03, 36.36 × 10^−6^ mol L^−1^ for curves a → k, respectively; curve m shows the emission spectrum of butein only, c(butein) = 36.36 × 10^−6^ mol L^−1^; (**b**) Stern–Volmer plots for the fluorescence quenching of XO by butein at different temperatures.

Fluorescence spectroscopy analysis was further used to research the binding properties of butein with XO on account of its ability to provide information on the molecular level of the binding mode, binding constant, and intermolecular distance. As shown in Figure 4a, under 280 nm excitation wavelength, the fluorescence emission spectra of XO inhibited by different concentrations of butein were excited. XO displayed two strong fluorescence emissions at 340 nm and 405 nm wavelengths due to the presence of three intrinsic fluorophores on XO, including tryptophan, tyrosine, and phenylalanine [30]. Nevertheless, butein has no intrinsic fluorescence under the same experiment conditions. Besides, fluorescence intensity of XO is mainly attributed to tryptophan residues due to the low quantum yield of phenylalanine. The results showed that XO emission intensities decreased dramatically with increasing of butein concentrations, which indicated that butein directly interacted with XO and quenched its intrinsic fluorescence. 

Generally, the ground state complex caused the static quenching, while the collision between the fluorophore and the quencher lead to the dynamic quenching. Their excited-state lifetime was used to distinguish two modes of mechanism [31]. The Stern–Volmer equation was applied to calculate the *K_SV_* and *K*_q_ values with maximal emission at 340 nm in order to ascertain the mechanism of butein binding to XO, as follows [30]:

(1) $$\frac{F_{0}}{F}\;=1\;+\;K_{SV}\left[Q\right]\;=\;1\;+\;K_{q}\tau_{0}\left[Q\right]$$

*F*_0_ and *F* are steady-state fluorescence intensities of fluorophore in the absence and presence of butein, respectively. *K_SV_* and [Q] are Stern–Volmer dynamic quenching constant and quencher concentration, respectively. *K*_q_ is the quenching rate constant of biomolecule (*K*_q_ = *K_SV_*/*τ*_0_). *τ*_0_ is the average biomolecule lifetime, and its value is 10^−8^ s [32]. *K_SV_* can be obtained from linear regression plot slope of *F*_0_/*F* versus [Q].

As shown in Figure 4b, the plots of *F*_0_/*F* versus [Q] at three temperatures (298, 304 and 310 K), and the relevant *K*_q_ values were given in Table 2. A high linearity suggested that only one type of quenching process occurred, either static or dynamic quenching. When the fluorophore is a biomacromolecule, its maximum scatter collision quenching constant is 2.0 × 10^10^ L mol^−1^ s^−1^ [33]. The *K*_q_ values (at 298, 304 and 310 K, respectively) were 2.09 × 10^12^, 2.55 × 10^12^, 3.10 × 10^12^ L mol^−1^ s^−1^, which were considerably greater than the maximum scatter collision quenching constant. The results implied that the fluorescence quenching mechanism of XO by butein is static quenching, and resulted from the formation of a butein–XO complex [34].

Afterwards, the fluorescence titration data were further analyzed to acquire the association constant (*K*_a_) applying the modified Stern–Volmer equation [35]:

(2) $$\frac{F_{0}}{F_{0}-F}=\frac{1}{f_{a}k_{a}\left[Q\right]}+\frac{1}{f_{a}}$$

where *f*_a_ is the fraction of accessible fluorescence. The linear regression of *F*_0_/(*F*_0_ − *F*) versus 1/[Q] gave the *K*_a_ values for the butein-XO complex at 298, 301, and 310 K (Table 2). The *K*_a_ values were in order of 10^4^ L mol^−1^ and the high linear correlation coefficient R indicated a moderate affinity for the butein–XO interaction [26].

For the static quenching reaction, it was assumed that small molecules bind independently into a class of equivalent sites on a macromolecule. The apparent binding constant *K*_b_ and the number of binding sites *n* can be calculated from the following equation [36]:

(3) $$\mathrm{lg}\frac{F_{0}-F}{F}\;=\;n\mathrm{lg}\;K_{b}\;+\;n\mathrm{lg}\left[Q\right]$$

where [Q] is the total concentrations of butein. As shown in Table 2, the values of *n* were found to be nearly equal to 1, implying the existence of one binding site for butein on XO, which was consistent with the results of inhibition-mode studies.

### 2.6. Thermodynamic Analysis

Generally, hydrogen bonds, van der Waals forces, electrostatic forces and hydrophobic interactions are regarded as the main forces acting small ligands bind to biomolecules. The acting force of the butein–XO complexation could be illustrated by calculating the thermodynamic parameters. The values of ∆H and ∆S were determined from the van’t Hoff equation:

(4) $$\mathrm{lg}K_{a}=-\frac{\Delta H}{2.303\mathrm{RT}}+\frac{\Delta S}{2.303R}$$

(5) ΔG= ΔH−TΔS

where *K*_a_ and *R* represent the binding constant and the gas constant (8.314 J mol^−1^ K^−1^). The temperatures were 298, 304 and 310 K. The values of ∆H and ∆S were determined by plotting log*K*_a_ versus 1/T, and then the free energy change (∆G) was obtained from Equation (5). As shown in Table 2, the negative signal for ∆G meant that the interaction of butein with XO was spontaneous. The positive values of ∆H (103.45 kJ mol^−1^) and ∆S (427.94 J mol^−1^ K^−1^) suggested that the binding was an exothermic process, and the complexation was predominately driven by hydrophobic forces [37].

### 2.7. Synchronous Fluorescence

**Figure 5:**
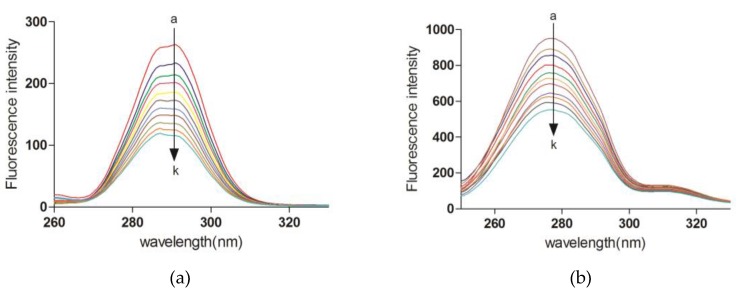
Synchronous fluorescence spectra of XO in the absence and presence of butein c(XO) = 0.1 U mL^−1^, and c(butein) = 0, 3.96, 7.84, 11.65, 15.39, 19.05, 22.64, 26.17, 29.63, 33.03, 36.36 × 10^−6^ mol L^−1^ for curves a → k, respectively. (**a**) ∆λ = 15 nm; (**b**) ∆λ = 60 nm.

The synchronous fluorescence spectroscopy has been performed to explore the molecular environment around the chromophore groups (tyrosine and tryptophan) of XO [38]. Both of the synchronous fluorescence intensities of tyrosine (∆λ = 15 nm) and tryptophan (∆λ = 60 nm) residues declined obviously with the addition of butein to XO solution. As shown in Figure 5, tyrosine had a visible blue-shift (from 290 nm to 286 nm) and tryptophan had a slight blue-shift (from 276 nm to 275 nm), suggesting that the polarity decreased and the hydrophobicity increased in the vicinity of tyrosine and tryptophan, specifically, that the fluorophores of XO were exposed in a less hydrophilic environment, and more exposed to the solutions with the addition of butein, which was consistent with the result of thermodynamic analysis.

### 2.8. Computational Docking of the Butein–XO Complex

**Figure 6:**
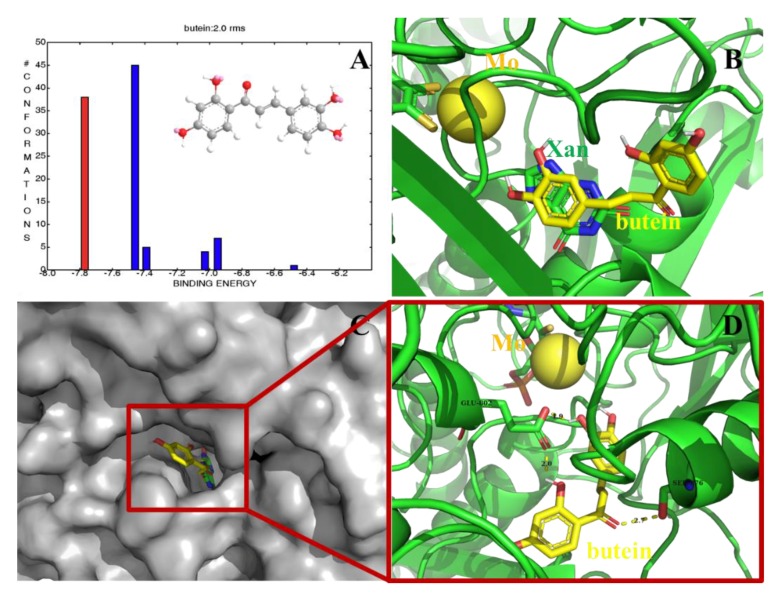
Molecular docking results of butein to XO. (**A**) Cluster analyses of the AutoDock docking runs of butein with 3EUB. (**B**,**C**) The binding active positions of xanthine (Xan) and butein with 3EUB. (**D**) Analysis of docked butein bound to 3EUB showing the key interactions in the binding pocket.

Molecular docking was carried out to improve the understanding of interactions between butein and XO protein. The crystal structure of XO enzyme (PDB ID, 3EUB) in complex with xanthine (Xan) from bovine milk was used for the docking calculations [39]. For validation of the credible docking method, the natural ligand Xan was docked back into the 3EUB protein as a control until the root mean square deviation (RMSD) of best docked ligand conformation was less than 2.0 Å. As shown in Figure 6A, a total of six conformational clusters were obtained from 100 docking runs at an RMSD tolerance of 2.0 Å, and the lowest binding energy with −7.77 kcal mol^−1^,were the most optimal among all the clusters (38 out of 100, red histogram in Figure 6A). In particular, as shown in Figure 6B,C, ligand Xan and potent inhibitor butein were located at the same hydrophobic pocket of XO, which is the active site with molybdenum atomic (Mo) domain, indicating that butein is a competitive xanthine oxidase inhibitor against xanthine with occupying the catalytic center of XO to avoid the entrance of xanthine and inducing conformational changes in XO. The docking results for butein suggest that three hydrogen bonds were formed between the OH group of butein and the oxygen or hydrogen atom of key amino acids (Figure 6C,D). The carboxy group of Glu802 was found to form H-bonds with the hydrogen atom of 3-OH and 2′-OH in butein respectively. The carbonyl group in butein was connected with the hydrogen atom of *β*-OH in Ser876. In addition, key hydrophobic contacts of residues Ala1079, Phe914, Phe1009, Ala1078, Arg880, Val1011, Leu1014, Leu873 and Leu648 were also found in the hydrophobic region of XO. All of these suggest that hydrogen bonds and hydrophobic interaction play an important role in the binding between butein and XO.

## 3. Experimental Section

### 3.1. Materials

Mass spectra were obtained with a Bruk micro-TOFQ mass spectrometer. NMR spectra were acquired on a Bruker AV-500 FT-NMR spectrometer (Bruker Daltonics, Bremen, Germany) operating at 500.1 MHz for ^1^H and at 125.8 MHz for ^13^C at 25 °C. The chemical shifts were referenced to the residual solvent signals *δ*_H_ 2.05 and *δ*_C_ 206.2, 29.8 for (CD_3_)_2_CO. 2,4-dihydroxyacetophenone and 3,4-dihydroxybenzaldehyde were purchased from Aladdin Chemical Reagent Company (Shanghai, China). Xanthine oxidase from bovine milk (0.2 units/mg protein), whereas xanthine, sodium phosphate, allopurinol were purchased from Sigma and Aldrich Chemical Co. (St. Louis, MO, USA).

### 3.2. General Procedure for the Synthesis of Butein

SOCl_2_ (0.4 mL) was added dropwise to a stirred solution of the 2,4-dihydroxyacetophenone (1 mmol) and 3,4-dihydroxybenzaldehyde (1 mmol) in ethanol (5.1 mL). The reaction mixture was stirred at room temperature for 2 h. At the end of reaction, the mixture was extracted with ethyl acetate by the addition of water. The ethyl acetate layer was washed with water and saturated brine, dried over anhydrous Na_2_SO_4_. After concentration under reduced pressure, the crude product was further purified by reversed-phase preparative HPLC (Agilent Prepstar SD-1 pump connected to a Prostar UV-Vis detector, column Megress ODS-C_18_, 20 × 250 nm, i.d., 10 μm) to give butein. The elution was MeOH-H_2_O (55:45, *v*/*v*) at a flow rate 3.0 mL/min, and the sample injection volume was 200 μL. The UV detector was set at 254 and 365 nm.

*[1-(2,4-Dihydroxyphenyl)-3-(3,4-dihydroxyphenyl) prop-2-en-1-one]* (**c**): Yellow power, ^1^H NMR (acetone-*d*_6_) *δ*: 13.62 (1H, s, 2′-OH), 8.11 (1H, d, *J* = 8.8 Hz, H-6′), 7.77 (1H, d, *J* = 15.2 Hz, H-*β*), 7.68 (1H, d, *J* = 15.2 Hz, H-*α*), 7.35 (1H, br s, H-2), 7.22 (1H, d, *J* = 8.1 Hz, H-6), 6.91 (1H, d, *J* = 8.1 Hz, H-5), 6.47 (1H, dd, *J* = 8.8, 1.8 Hz, H-5′), 6.37 (1H, d, *J* = 1.8 Hz, H-3′). ^13^C NMR (acetone-*d*_6_) *δ*: 192.8 (C=O), 167.6 (C-4′), 165.6 (C-2′), 149.3(C-4), 146.5 (C-3), 145.5 (C-*β*), 133.3 (C-6′), 128.2 (C-1), 123.4 (C-6), 118.3 (C-*α*), 116.4 (C-2), 116.0 (C-5), 114.5 (C-1′), 108.7 (C-5′), 103.8 (C-3′). ESI-MS *m*/*z* 273.08, [M + H]^+^.

### 3.3. Enzyme Activity Assay

The XO inhibitory activity assay was performed according to the method modified by our group [29]. The butein was dissolved in dimethyl sulfoxide (DMSO) and subsequently diluted with phosphate buffer (0.07 mol L^−1^, pH = 7.5) to a final concentration containing less than 1% DMSO (*v*/*v*). In the 0.8 mL reaction system, a series of assay solutions prepared in phosphate buffer (0.07 mol L^−1^, pH = 7.5) consisting of a fixed concentration of XO (0.024 U mL^−1^) and various concentrations of inhibitor solutions were incubated at 25 °C for 30 min, and the reaction was initiated by adding the substrate xanthine (300 × 10^−6^ mol L^−1^) to the complex solutions. The assay mixture was incubated at 25 °C for 30 min. The reaction was stopped by the addition of HCl (1 mol L^−1^). The absorbance of uric acid level was determined by an HPLC method using a Diamonsil ODS-C_18_ column (250 × 4.6 mm i.d., 5 μm, Dikma Technologies, Beijing, China) on an Agilent 1260 system equipped with a G1311C quaternary pump, a G1329B autosampler, a G1316A thermostatted column compartment, and a G1314F variable wavelength detector coupled with an analytical workstation (Agilent Technologies, Inc., Santa Clara, CA, USA). Averages of three replicates are presented and the allopurinol was used as a positive control.

### 3.4. Kinetic Analysis for Inhibitory

The assay was investigated applying the XO activity assay methodology in the absence and presence of butein with a series of concentrations of substrate xanthine (25, 37.5, 50, 75, and 100 × 10^−6^ mol L^−1^), where butein was at diverse concentrations (0, 0.2, 0.4, 0.8 × 10^−6^ mol L^−1^) and xanthine at a fixed concentration. The involved inhibition mode of the butein on xanthine oxidase was analyzed from Lineweaver–Burk plots, and *K*_i_ value was identified by the software GraphPad Prism 5.0 (GraphPad Software Inc., La Jolla, CA, USA). For competitive inhibition, the Lineweaver–Burk equation can be written in double reciprocal form as follows:

(6) $$\frac{1}{v}=\frac{K_{m}}{V_{\max}}\left(1+\frac{\left[I\right]}{K_{i}}\right)\frac{1}{\left[S\right]}+\frac{1}{V_{\max}}$$

A secondary plot can be plotted from:

(7) $$K_{m}{}^{\mathrm{app}}=\frac{K_{m}\left[I\right]}{K_{i}}+K_{m}$$

where *K*_i_ and *K*_m_ represent the inhibition constant and Michaelis–Menten constant, respectively, and their values can be calculated from Equations (6) and (7). *v* denotes enzyme reaction rate in the absence and presence of butein. [I] and [S] are the concentrations of the inhibitor and substrate, respectively. *K*_m_^app^ presents the apparent Michaelis-Menten constant. The secondary plot of *K*_m_^app^ versus [I] is linearly fitted, indicating a single inhibition site or a single class of inhibition site [40].

### 3.5. Fluorescence Titration

Fluorescence titration assay was conducted by a spectrofluorimeter (Model F-4600, Hitachi, Japan) equipped with a thermostat bath and a 150 W xenon lamp to characterize the interaction between butein and XO. Briefly, 2.0 mL sample of XO solution (0.1 U mL^−1^) was titrated with different concentrations of butein from 0 to 36.36 × 10^−6^ mol L^−1^ and then the solutions were incubated 5 min to equilibrate. Afterwards, fluorescence emission spectra of the solutions were measured using a spectrofluorometer at different temperatures (298, 304 and 310 K) in 300–500 nm wavelength range upon excitation 280 nm. The widths of both the excitation and emission slits were set at 5 nm. The background fluorescence of the buffer (0.07 mol L^−1^ PBS solution, pH = 7.5) was subtracted from butein–XO complexes.

Synchronous fluorescence spectra were performed by setting the excitation and emission wavelength interval (∆λ) at 15 and 60 nm over a wavelength range of 260–330 nm and 250–330 nm, respectively.

All the fluorescence-quenching data were corrected for absorption of excitation light and reabsorption of emitted light by the following relationship [41]:

*F* = *F*_m_*e*^(A1 + A2)/2^ (8)

where *F*_c_ and *F*_m_ represent the corrected and measured florescence. A_1_ and A_2_ are the absorbance of butein at excitation and emission wavelengths, respectively.

### 3.6. Molecular Docking

The potential binding site of XO-butein was researched on AutoDock (version 4.2, The Scripps Research Institute, La Jolla, CA, USA). The X-ray crystal structure (PDB ID, 3EUB) of XO used for the docking studies [42] was download from the Protein Data Bank (http://www.rcsb.org/pdb). All water molecules and ligand in XO were removed prior to the docking program running, after which the polar hydrogen atoms and Gasteiger charges were added to the macromolecule file. The 3D structures of butein were obtained in Chem 3D Ultra 8.0. The active site with a fixed grid spacing (0.375 points) was enclosed by a rigid dimension of docking center (100 × 100 × 100 points). The best scoring docked model of the ligand was chosen to represent its most desirable binding mode predicted by AutoDock. The PyMol molecular graphic system was used to visualize the conformations and interactions between the butein and the target proteins.

## 4. Conclusions

In summary, a simple and effective method for the synthesis of butein by aldol condensation with the catalytic system SOCl_2_/EtOH was conducted for the first time in the present study. The optimal reaction conditions including the molar ratio of reactants, the dosage of catalyst, and the reaction time were investigated, and its potential inhibition of XO was discussed in aspect of inhibition kinetics, fluorescence titrations, synchronous fluorescence spectroscopy, and molecular docking. The butein can be prepared in good yield from 2′,4′-dihydroxyacetophenone and 3,4-dihydroxybenzaldehyde based on the catalytic system SOCl_2_/EtOH, without protecting the hydroxyl groups. In addition, the inhibitory activity on XO showed that butein could significantly reduce uric acid concentration and the IC_50_ value of restraining XO was found as 2.93 × 10^−6^ mol L^−1^ in vitro. Additionally, an attractive inhibition mechanism of irreversible competition is worth further exploration, as it might be helpful in treating gout. The intrinsic fluorescence of XO was quenched statically by butein, and butein bound spontaneously into the active cavity of XO to form the butein–XO complex with one high-affinity binding sites. Besides, hydrophobic interaction is the main driving force of butein to binding with XO. The molecule docking revealed butein is a competitive XO inhibitor and has a high affinity binding site on XO. The effectiveness of butein in reducing uric acid concentration in the blood is required to evaluate by the further in vivo studies. This study has supplied significant insights into the inhibitory mechanism of butein on XO, which will promote butein to be a leading compound by structural modification on the treatment of gout.

## Figures and Tables

**Table 1:** Optimization of parameters for the synthesis of butein.

Entry	SOCl_2_ (mol L^−1^)	Molar Ratio (a/b)	Time (h)	Yield (%)
1	0.25	1:1	2	2
2	0.5	1:1	2	74
3	1	1:1	2	85
4	1.5	1:1	2	37
5	2	1:1	2	9
6	1	1:0.6	2	50
7	1	1:0.8	2	60
8	1	1:1.2	2	75
9	1	1:1.4	2	76
10	1	1:1	0.5	84
**11**	**1**	**1:1**	**1**	**88**
12	1	1:1	1.5	87

**Table 2:** Quenching rate constant *K*_q_, modified Stern–Volmer association constant *K*_a_, and relative thermodynamic parameters for the interaction between butein and XO at different temperatures.

T (K)	*K*_q_ (× 10^12^ L mol^−1^ S^−1^)	R^a^	*K*_a_ (× 10^4^ L mol^−1^)	R^b^	∆H (kJ mol^−1^)	*n*	∆G (kJ mol^−1^)	∆S (J mol^−1^ K^−1^)
298	2.09	0.9953	2.96	0.9921	103.45	0.91	−24.09	427.94
304	2.55	0.9878	3.86	0.9931		0.75	−26.66	
310	3.10	0.9944	10.51	0.9991		1.00	−29.23	
